# Mounier–Kuhn syndrome: a tripartite analysis bridging clinical epidemiology, imaging evolution, and global research landscapes

**DOI:** 10.1186/s13023-025-03745-w

**Published:** 2025-05-19

**Authors:** Yanjie Wang, Luyao Wang, Haoxiang Zhang, Xiaojia Zhu, Xiaoxi Shi, Sirui Fu, Kai Sun, Jiao Wang, Quanwei Ren, Yongjin Ji, Changqing Zhao

**Affiliations:** 1 Department of Otolaryngology Head and Neck Surgery, Second Hospital of Shanxi Medical University, 382 Wuyi Road, Taiyuan, 030000 Shanxi Province China; 2 Key Research Laboratory of Airway Neuroimmunology in Shanxi Province, Taiyuan, China; 3 Department of Radiology, Second Hospital of Shanxi Medical University, Taiyuan, China; 4 Department of Respiratory and Critical Care, Second Hospital of Shanxi Medical University, Taiyuan, China

**Keywords:** Mounier–Kuhn syndrome, Tracheobronchomegaly, Chronic respiratory infections, Cough, Management

## Abstract

**Background:**

Mounier–Kuhn syndrome (MKS) is characterized by tracheobronchomegaly with thinning or atrophy of the elastic tissue. Due to low clinical awareness, MKS is frequently overlooked on chest CT examinations, leading to diagnostic delays. This study aimed to synthesize the historical context and contemporary advancements in MKS research.

**Methods:**

Five MKS cases were retrospectively identified through thoracic imaging review at our institution. A systematic review adhering to PRISMA guidelines was conducted across Web of Science (WOS) and China-specific databases (China National Knowledge Infrastructure [CNKI], Wanfang) from January 2000 to March 2025 to identify studies reporting CT-confirmed tracheobronchial dilation, to address geographic bias. Concurrently, a bibliometric analysis of WOS publications spanning January 1962 to March 2025 was performed using predefined inclusion criteria to analyze historical research trends through VOSviewer.

**Results:**

Our institutional cohort (5 patients: 4 males) exhibited marked tracheobronchial dilation, with two representative cases demonstrating distinct clinical trajectories of disease progression. Systematic analysis of 147 publications encompassing 169 radiologically confirmed cases revealed significant male predominance (male-to-female ratio: 5.5:1), a mean tracheal diameter of 34.3 ± 6.1 mm, a median diagnostic delay of 3.0 years (IQR: 0.25–20.0 years), and high comorbidity prevalence including bronchiectasis (71.6%) and tracheal diverticulosis (67.5%). The most frequent clinical manifestations were cough (64.5%), dyspnoea (52.7%), and recurrent respiratory infections (57.4%). Bibliometric analysis of 288 global publications characterized research trends through country/institutional affiliations, author collaborations, journal distributions, and keyword co-occurrence, with diagnostic imaging advancements dominating recent scholarly output.

**Conclusions:**

This three-phase analytical approach bridges clinical observations with global research trends, revealing significant diagnostic delays and evolving imaging paradigms in MKS management. Our findings underscore the need for enhanced clinical vigilance and multinational collaborative research initiatives to establish evidence-based therapeutic frameworks for this under-diagnosed condition.

**Supplementary Information:**

The online version contains supplementary material available at 10.1186/s13023-025-03745-w.

## Introduction

Mounier–Kuhn syndrome (MKS), also known as tracheobronchomegaly (TBM), is an uncommon congenital condition characterized by abnormal airway dilation [1]. Histological changes in enlarged airways were first described through autopsy by Czyhlarz in 1897 [2]. Later, in 1932, Mounier–Kuhn discussed the correlation between endoscopic and radiographic findings [3]. Fewer than 500 cases have been reported worldwide, with incidences ranging from 0.5% to 1.6%.[4,5]Patients who are asymptomatic are often misdiagnosed or undiagnosed. Therefore, the actual number of cases may be much greater. In 2016, Eduards Krustins conducted a systematic review of literature published on MKS within the last 25 years [4]. The number of case reports published since then is substantial, and scientific advancements are offering a variety of new diagnostic and treatment options for these patients. Accordingly, we conducted a detailed and systematic analysis of MKS by combining the clinical data from 5 case reports of MKS with a bibliometric review of 288 related articles. This study provides a systematic synthesis of the published MKS literature to date, integrating multisource data from institutional imaging, a systematic case review, and bibliometric trends.

## Methods

This study employed a two-pronged approach combining clinical case analysis with systematic literature review. For the clinical evaluation, five patients who were diagnosed with MKS at our institution between September 2010 and August 2024 were retrospectively analysed. The diagnosis was confirmed through chest CT imaging via the following established criteria: tracheal diameters exceeding 25.0 mm (coronal) and 27.0 mm (sagittal) in males, and 21.0 mm (coronal) and 23.0 mm (sagittal) in females, with corresponding main bronchus measurements as per international guidelines [5–7].

For a comprehensive literature assessment, we conducted systematic searches across three major databases: the Web of Science(WOS) Core Collection (including the Science Citation Index-Expanded and Social Sciences Citation Index), the China National Knowledge Infrastructure (CNKI), and the Wanfang Medical Database. The search strategy utilized MeSH terms and Boolean operators: (“Mounier–Kuhn syndrome” OR “tracheobronchomegaly” OR “tracheomegaly” OR “bronchomegaly”). The systematic search encompassed publications from January 2000 to March 2025, ensuring comprehensive coverage of the defined temporal scope. Two independent researchers performed article screening using predefined inclusion criteria: (1) original research or case reports with diagnostic confirmation, (2) English or Chinese language publications, and (3) adult patient data (≥ 18 years). The exclusion criteria included editorials, conference abstracts, and studies lacking quantitative measurements. Discrepancies were resolved through consensus discussion with a senior investigator. The data extracted included demographic characteristics, diagnostic methods, treatment modalities, and clinical outcomes. Statistical analysis of the clinical data was performed with SPSS (version 27.0), with continuous variables expressed as the means ± standard deviations and categorical variables expressed as frequencies.

Our bibliometric analysis included MKS-related publications from January 1962 to March 2025, with differential processing for English and Chinese databases. English-language publications (WOS) were subjected to comprehensive network analysis via VOSviewer 1.6.20 to map research trends, author collaboration clusters, and keyword co-occurrence patterns. Chinese-language literature (CNKI/Wanfang) was analysed exclusively for temporal publication trends due to its limited therapeutic innovation content. This dual-language approach balanced global research dynamics with region-specific scholarly output characteristics.

## Results

Between 2010 and 2024, five patients (4 males, 1 female) with MKS were retrospectively identified at the Second Hospital of Shanxi Medical University through diagnostic imaging archives (Table 1). Two clinically distinct cases were selected to demonstrate the phenotypic spectrum of MKS, emphasizing variations in disease progression and therapeutic management.

**Table 1:** Clinical characteristics, airway morphology, and management outcomes of MKS cases (n = 5)

Cases	Age (years)/Sex	Diagnostic Delay (years)	Chief complaints	Transverse/sagittal diameter of the trachea (mm)	Right/left main bronchus diameter (mm)*	Treatment plan	Follow-up (months)/Outcome
Case 1	86/Male	20	Chronic Cough, fever, Purulent Sputum	42.1/43.5	18.0/14.0	Antimicrobial and expectorant therapy	1/death, multiorgan failure
Case 2	65/Male	0.25	Chronic Cough, Purulent Sputum	33.8/35.5	21.3/18.9	Antimicrobial and expectorant therapy	12/stable
Case 3	46/Male	2	Chronic cough	37.5/38.6	22.0/19.0	Corticosteroids and bronchodilator aerosols	60/stable
Case 4	63/Female	37	Intermittent wheezing	23.3/18.3	23.8/32.2	Antimicrobial and expectorant therapy, intensive respiratory therapy, corticosteroids and bronchodilator aerosols	60/stable
Case 5	73/Male	10	Dyspnoea, Chronic Cough, Purulent Sputum	29.5/31.5	22.3/24.6	Antimicrobial and expectorant therapy, intensive respiratory therapy	24/3 hospitalizations

Abbreviations: MKS: Mounier–Kuhn syndrome

*Right/left bronchial transverse diameters were obtained from coronal CT images, which is consistent with the diagnostic criteria of Krustins et al. [4]

### Case report

#### Patient illustration 1 (case 1)

**Fig. 1:**
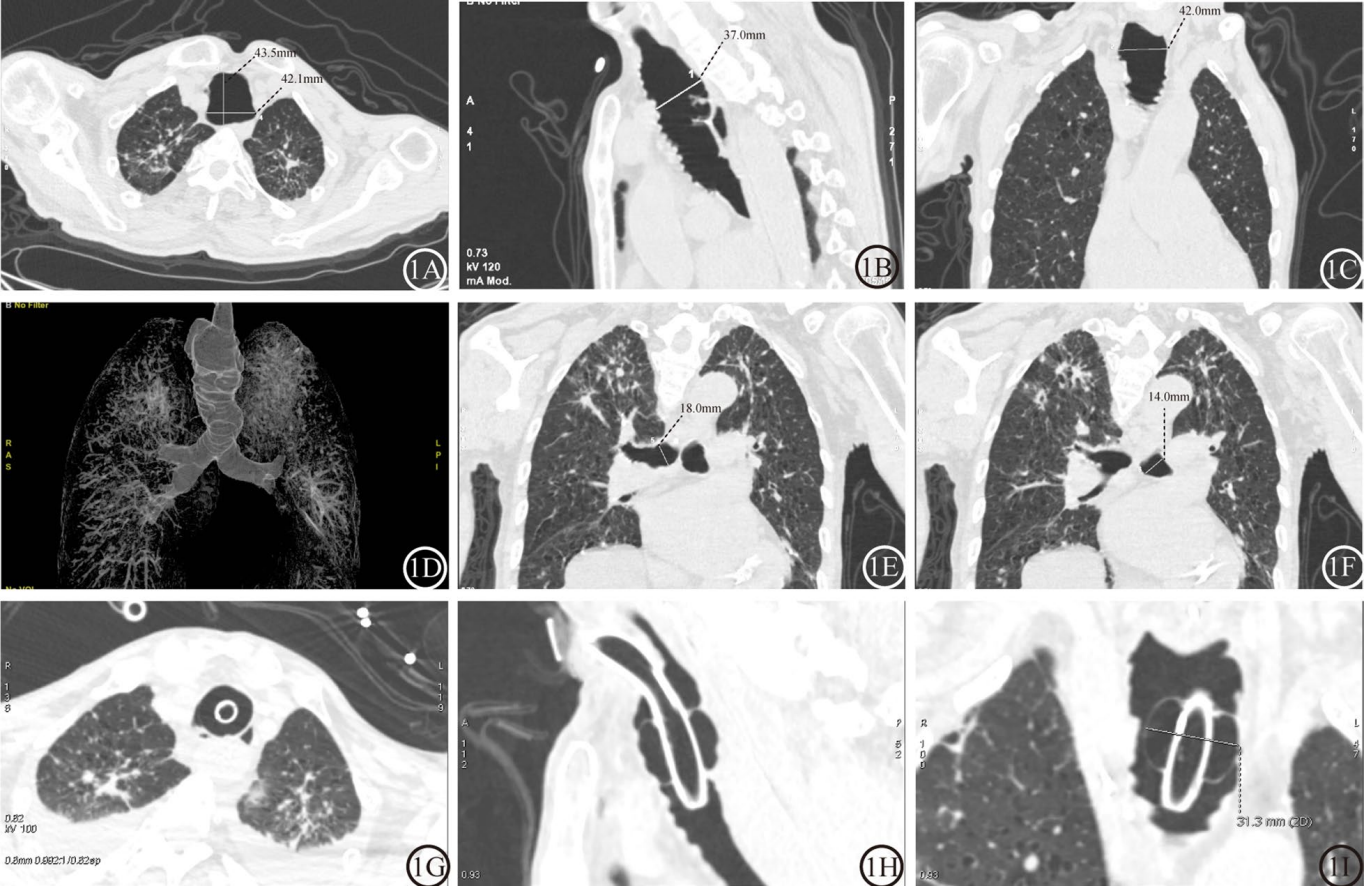
Multimodal CT evaluation of MKS (Case 1). **A**–**C** Axial, sagittal, and coronal views demonstrating pantracheobronchial dilation. **D** 3D airway reconstruction reveals main bronchial dilatation with pathognomonic features: multiple diverticula and cystic bronchiectasis. **E**, **F** Coronal oblique MIP images confirming bilateral mainstem bronchus dilation. **G**–**I** Posttracheostomy CT images revealing an inadequate cuff seal with air leakage, measured at the residual tracheal lumen.Abbreviations: MKS: Mounier–Kuhn syndrome; MIP, Maximum Intensity Projection; CT, computed tomography

An 86-year-old man presented with acute-onset delirium, hypersomnolence, and fever persisting for 7 days, superimposed on a two-decade history of recurrent productive cough. He had a 40 pack-year smoking history (cessation 30 years prior). Three-dimensional CT airway reconstruction revealed diffuse tracheobronchomalacia with pantracheobronchial diverticulosis and cystic bronchiectasis, generating a pathognomonic cobblestone morphology (Fig. 1A–F). Bronchoscopy confirmed tracheal dilation with mucosal inflammation but no congenital anomalies. Progressive respiratory failure necessitated tracheostomy, which was complicated by ventilator air leakage due to an inadequate cuff seal (Fig. 1G–I). A multidisciplinary consensus was used to diagnose MKS with refractory *Pseudomonas aeruginosa* pneumonia. Despite receiving meropenem therapy guided by antimicrobial susceptibility testing, the patient showed no significant clinical improvement. Ultimately, the patient's family opted to withdraw life-sustaining treatment after comprehensive counseling regarding the patient's prognosis and therapeutic alternatives. During the subsequent 10-day follow-up period, the patient developed acute respiratory failure and succumbed.

#### Patient illustration 2 (case 4)

**Fig. 2:**
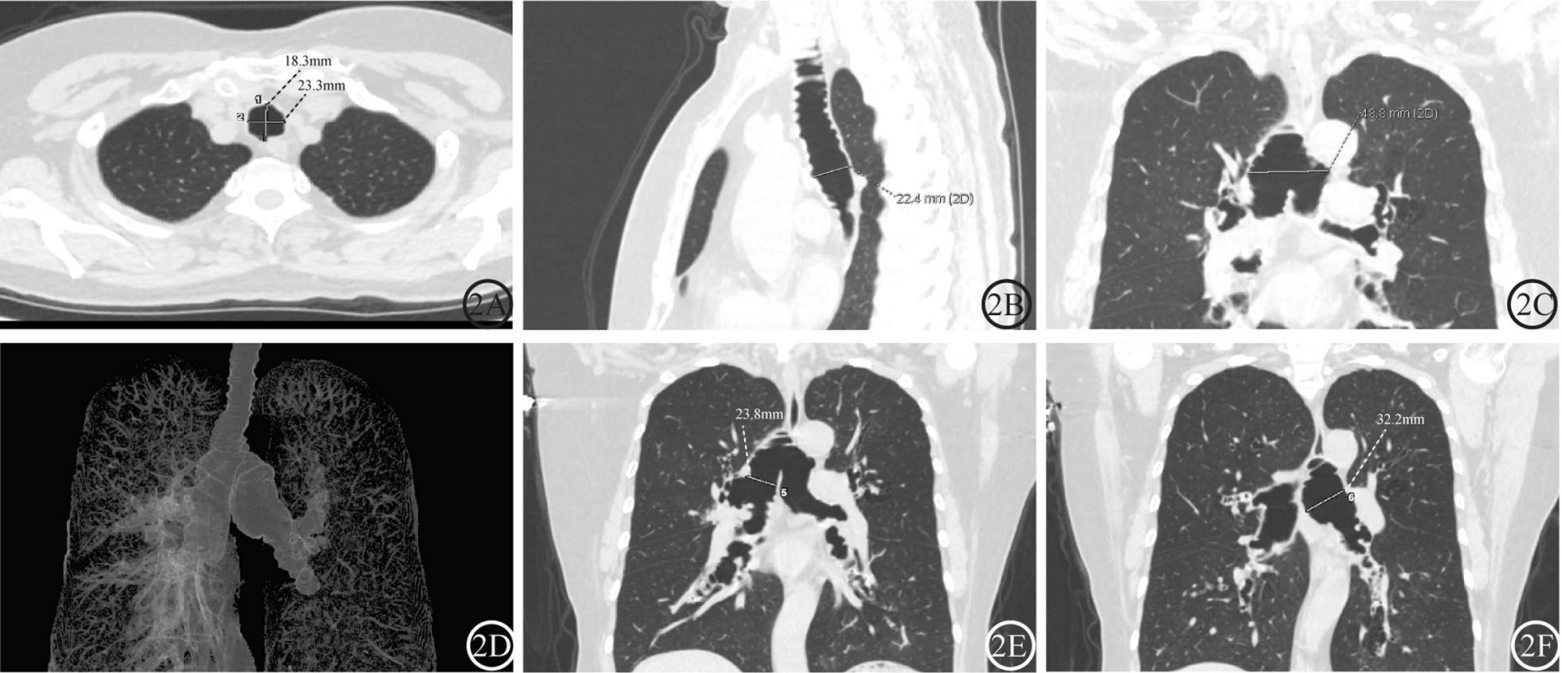
Multimodal CT evaluation of MKS (Case 4). **A**–**C** Axial, sagittal, and coronal views demonstrating pantracheobronchial dilation. **D** 3D airway reconstruction reveals main bronchial dilatation with pathognomonic features: multiple diverticula and cystic bronchiectasis. **E**, **F** Coronal oblique MIP images confirming bilateral mainstem bronchus dilation. Abbreviations: MKS: Mounier–Kuhn syndrome; MIP, maximum intensity projection; CT, computed tomography

A 63-year-old woman was referred for pulmonology evaluation due to progressive exertional wheezing. She was initially diagnosed with asthma at age 26, with seasonal exacerbations characterized by purulent sputum production. She denied tobacco use. High-resolution CT revealed tracheobronchomegaly, bilateral emphysematous bullae, and left lower lobe consolidation (Fig. 2A–F). Multidisciplinary assessment reclassified the diagnosis as MKS with an overlapping asthma-COPD phenotype. The implementation of a regimen combining high-frequency chest wall oscillation, tailored antibiotic therapy (guided by sputum cultures), inhaled corticosteroids, and long-acting β2-agonists achieved clinically significant improvement, with sustained stability at the 12-month follow-up.

### Systematic review of the literature from 2000 to 2025

Our systematic review identified 169 MKS cases (male predominance: 84.6% [143/169]; age: 55.9 ± 16.6 years [mean ± SD], range 23–94 years) with marked tracheobronchial dilation (tracheal diameter: 34.3 ± 6.1 mm [mean ± SD], range 22.0–57.0 mm) (Table 2). A 5.5:1 male-to-female ratio and geographic heterogeneity were observed, with 12.4% (21/169) of cases reported in Europe, 29.6% (50/169) in North America, and 31.4% (53/169) in China. Anatomical analysis revealed tracheal diverticulosis in 67.5% (114/169) of the patients and bronchiectasis in 71.6% (121/169). Clinically, chronic cough was documented in 64.5% (109/169) of patients (productive subtype: 44.4% [75/169]), recurrent infections in 57.4% (97/169), and dyspnoea in 52.7% (89/169). Haemoptysis occurred less frequently (14.2%, 24/169).

**Table 2:** Characteristics of 169 Cases (from 147 Publications)

	Females 26 (15.4%)	Males 143 (84.6%)	In total 169 (100%)
Reporting hospital (region)			
Europe	15.4% (4)	11.9% (17)	12.4% (21)
North America	23.1% (6)	30.8% (44)	29.6% (50)
China	30.8% (8)	31.5% (45)	31.4% (53)
Other	30.8% (8)	25.9% (37)	26.6% (45)
Age, years			
Mean ± SD	55.2 ± 18.1	56.1 ± 16.4	55.9 ± 16.6
Youngest	23	25	23
Oldest	91	94	94
Smokers, %(n)			
Former/current	3.8% (1)	36.4% (52)	31.4% (53)
Never	76.9% (20)	26.6% (38)	34.3% (58)
No data	19.2% (5)	37.1% (53)	34.3% (58)
Average tracheal diameter, mm (Mean ± SD)	33.5 ± 6.3	34.4 ± 6.1	34.3 ± 6.1
Smallest	22.0	25.0	22.0
Largest	46.7	57.0	57.0
Average bronchial diameter, mm			
Left bronchus (Mean ± SD)	23.2 ± 7.4	23.6 ± 5.8	23.5 ± 6.0
Right bronchus (Mean ± SD)	24.9 ± 6.2	24.5 ± 5.4	24.6 ± 5.5
No data,%(n)cases	46.2% (12)	40.6% (58)	41.4% (70)
Tracheal diverticulosis noted in %(n)cases	76.9% (20)	65.7% (94)	67.5% (114)
Bronchiectasis noted in %(n)cases	80.8% (21)	69.9% (100)	71.6% (121)
Chief complaints			
Cough	50.0% (13)	67.1% (96)	64.5% (109)
Dry	3.8% (1)	23.1% (33)	20.1% (34)
Productive	46.2% (12)	44.1% (63)	44.4% (75)
Recurrent respiratory infections	46.2% (12)	59.4% (85)	57.4% (97)
Haemoptysis	7.7% (2)	15.4% (22)	14.2% (24)
Dyspnoea	50.0% (13)	53.1% (76)	52.7% (89)
Changes upon auscultation (wheezes, bronchi, crepitation)	65.4% (17)	58.0% (83)	59.2% (100)
Fever	26.9% (7)	21.7% (31)	22.5% (38)
Diagnostic Delay, years (Median, IQR)	–	–	3.0, 0.25–20.0 (67)
Treatment plan			
Conservative treatment	76.9% (20)	69.2% (99)	70.4% (119)
Ventilator-assisted treatment	15.4% (4)	10.5% (15)	11.2% (19)
Surgery	3.8% (1)	11.2% (16)	10.1% (17)
No data, %(n) cases	3.8% (1)	9.1% (13)	8.3% (14)
Therapeutic effect			
Improved	11.5% (3)	7.7% (11)	8.3% (14)
Stable	69.2% (18)	61.5% (88)	62.7% (106)
Uncontrolled	7.7% (2)	8.4% (12)	8.3% (14)
No data,%(n)cases	11.5% (3)	22.4% (32)	20.7% (35)

Analysis of 67 MKS cases with explicit symptom-to-diagnosis documentation revealed a median diagnostic delay of 3.0 years (IQR: 0.25–20.00 years), reflecting extreme variability in diagnostic timelines. Stratified analysis revealed that 25% of cases achieved diagnosis within 3 months of symptom onset, whereas the upper quartile experienced prolonged delays surpassing two decades. Clinically, a predominant subset of patients suffered protracted courses of recurrent lower respiratory tract infections, persistent cough, and dyspnoea prior to definitive diagnosis, though longitudinal symptom documentation remained fragmented. This pronounced disparity between symptom emergence and diagnostic confirmation underscores a critical gap in clinical awareness of MKS phenotypes. Conservative management constituted the primary therapeutic modality (70.4%, 119/169), followed by ventilator-assisted respiratory support (11.2%, 19/169) and surgical intervention (10.1%, 17/169). Sex-stratified analysis revealed significantly higher surgical rates in males (11.2% [16/143] vs. 3.8% [1/26]; χ^2^ = 4.32, *p* = 0.038). Clinical stabilization was achieved in 62.7% (106/169) of the patients, with equivalent rates of symptomatic improvement (8.3%, 14/169) and treatment failure (8.3%, 14/169). Data completeness varied substantially across parameters (treatment documentation gap: 8.3% [14/169]; outcome reporting deficiency: 20.7% [35/169]).

### Bibliometric analysis from 1962 to 2025

**Fig. 3:**
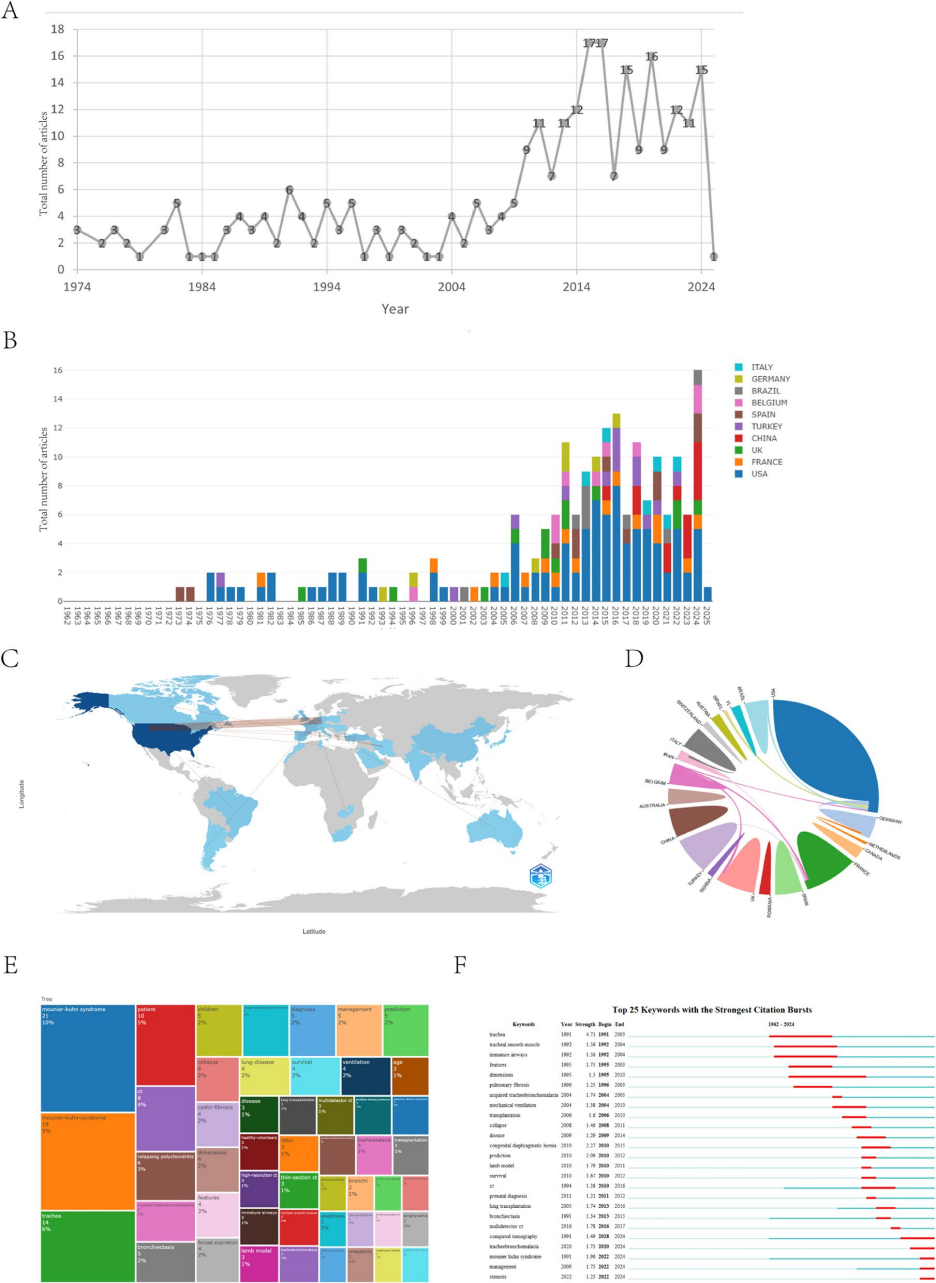
Bibliometric and visual analysis. **A** Temporal trends in English-language publications (Web of Science Core Collection). **B** Global publication density map (VOSviewer v1.6.19). **C** Annual contributions from the top 10 countries (threshold: ≥ 15 papers). **D** International collaboration network (minimum of 5 coauthored papers). **E** Keyword co-occurrence clusters. **F** Keyword bursts*. Note: *X-axis: Chronological span (1962–2025) delineating periods of sustained keyword prominence. Red bars represent high-strength citation bursts, indicating a dominant thematic focus. The blue bars correspond to moderate-strength bursts, reflecting emerging trends

A total of 288 articles related to MKS were included in this study. Figure 3A displays the annual count of English-language MKS publications from 1962 to 2025. Chinese-language publications exhibited delayed emergence, with 94.7% (54/57) published after 2010, as detailed in Supplementary Figure S1. Figure 3B shows the publication volume trends among the top ten productive countries. Figure 3C combines a global geographic distribution map of MKS research density with intercountry collaboration networks, whereas Fig. 3D specifically visualizes collaborative relationships between nations. A total of 377 institutions worldwide have contributed MKS-related publications, with the top eight institutions detailed in Supplementary Table S1. The three-field Sankey diagram (Supplementary Fig. S2) illustrates connections among countries, authors, and institutions. Keyword co-occurrence analysis (349 terms) is presented in Fig. 3E, and emerging research trends identified through keyword burst detection are shown in Fig. 3F. Publications were distributed across 132 journals, with the top 10 journals ranked by output listed in Supplementary Table S2. Three highly cited papers from the WOS database are catalogued in Supplementary Table S3.

## Discussion

As William Osler astutely remarked, “to study the phenomena of disease without books is to sail an uncharted sea, while to study books without patients is not to go to sea at all.” This axiom underscores the imperative integration of theoretical and clinical perspectives in medicine. Mounier–Kuhn's seminal 1932 report revolutionized our understanding of this condition through pioneering endoscopic and radiographic investigations, ultimately leading to its eponymous designation as MKS [3]. Comprehensive analysis of MKS thus requires dual examination of its clinical spectrum and historical diagnostic evolution, with bibliometric data demonstrating sustained global research productivity despite annual publication fluctuations.

MKS manifests as idiopathic tracheobronchial dilation with characteristic histopathological findings: atrophy of airway wall elastic fibres and smooth muscle layer hypoplasia [8]. Progressive parenchymal deterioration represents a hallmark pathophysiological feature of this disease [9], with symptom onset spanning 18 months to 84 years of age (peak incidence: 30–50 years). In our cohort, the mean age at diagnosis was 55.9 ± 16.6 years, consistent with previously reported epidemiological trends. Our findings confirm a striking male predominance (male-to-female ratio 5.5:1), aligning with epidemiological reports of ≈8:1 male-to-female disparities [10]. The etiopathogenesis remains debated, with two prevailing theories: (1) congenital origin via autosomal recessive inheritance, supported by autopsy evidence of tracheobronchial submucosal tissue deficiency [11]; and (2) acquired pathogenesis from prolonged mechanical ventilation-induced airway remodelling, particularly in premature infants [12]. Notably, our cases showed no familial clustering or comorbidities with connective tissue disorders (Ehlers–Danlos syndrome, Marfan syndrome), which are occasionally associated with MKS [13].

MKS exhibits marked interindividual variability in clinical presentation, with nonspecific respiratory manifestations predominating. Chronic cough (64.5%), productive sputum (44.4%), and progressive dyspnoea (52.7%) constitute the cardinal triad, whereas fever (22.5%) and haemoptysis (14.2%) represent less frequent features. Symptom onset typically emerges in the third decade,although paediatric-onset recurrent infections are documented. Notably, 8.3% of patients remain asymptomatic at initial diagnosis, although few maintain a lifelong symptom-free status. Frequent comorbid manifestations include bronchiectasis (71.6%) and recurrent pneumonia (57.4%). Emerging evidence suggests potential SARS-CoV-2-associated tracheomegaly progression, exemplified by a documented case of post-COVID-19 symptomatic exacerbation [14]. Extrathoracic associations encompass nasal polyposis and congenital craniofacial anomalies—bilateral ptosis, epicanthal folds, micrognathia, and upper lip redundancy—observed in syndromic variants [15]. Laryngeal involvement, manifesting as progressive hoarseness secondary to vocal cord paralysis or cricoarytenoid joint remodelling, has been mechanistically linked to tracheobronchial wall instability [16].

Typically, tracheal diameters exceeding sex-specific thresholds (coronal: males ≥ 25 mm, females ≥ 21 mm) remain pathognomonic [6, 17]. Posteroanterior chest radiography may reveal tracheal luminal expansion approximating the vertebral body width, which is more conspicuous in lateral projections. High-resolution computed tomography (HRCT) constitutes the diagnostic gold standard (see Methods) [18]. Three-dimensional CT reconstructions demonstrate pantracheobronchial dilation with characteristic saccular protrusions between cartilage rings, correlating bronchoscopically with dynamic mucosal herniation during forced expiration [19–21]. Alterations in the fluorescence of fibres have also been observed via confocal microscopy in patients with tracheobronchomegaly syndrome [8]. Autofluorescence imaging (AFI), when integrated with bronchoscopy, provides real-time visualization of tracheobronchial mucosal changes. Diminished AFI signal intensity in these regions correlates histopathologically with elastic fibre depletion or atrophy [22].

In summary, an MKS diagnosis hinges on the integration of characteristic clinical profiles and pathognomonic imaging criteria. The median diagnostic delay of 3.0 years (IQR: 0.25–20.0 years) and extreme interquartile variability in MKS suggest persistent underdiagnosis. Our case-driven insights position MKS as a critical differential in patients exhibiting chronic cough, sputum production, and haemoptysis alongside refractory lower respiratory infections. Severe manifestations may progress to exertional dyspnoea complicated by bronchiectasis-related haemoptysis [23], with rare presentations including spontaneous pneumothorax, life-threatening haemoptysis, and digital clubbing [24]. When conventional therapies fail in this context, multidisciplinary evaluation should prioritize dynamic airway assessment via forced-expiration CT or bronchoscopy to confirm the tracheobronchomalacia patterns. Standardized educational protocols integrating early HRCT evaluation of central airway dilation and recurrent pneumonia etiologies may mitigate diagnostic delays, especially in subclinical presentations.

The diagnostic workup must rigorously differentiate MKS from acquired tracheobronchial dilation and structural variants. Key exclusions include the following: (1) fibrotic tracheomegaly secondary to pulmonary fibrosis-induced opposing traction; (2) mucosal pseudodilation syndromes (laryngoceles, Zenker's diverticulum) lacking true airway wall pathology; and (3) apical lung herniations and bullous emphysema (airspace > 1 cm without tracheal involvement). Williams-Campbell syndrome is characterized by congenital cystic bronchiectasis resulting from a lack of cartilage in the fourth- to sixth-order bronchi [4, 25–30].

Postdiagnosis, a comprehensive evaluation of respiratory functional impairment grading and disease trajectory is critical for prognostication. Pulmonary function tests may reveal obstructive patterns with elevated residual volumes or they may remain normal [25]. In our cohort, spirometry demonstrated moderate obstruction in one patient and mild restriction in two other patients. Innovative assessment protocols, such as Pacheco’s single-breath nitrogen washout method, address MKS-specific challenges by quantifying anatomical dead space [9]. The nosological classification of MKS remains contentious, with two prevailing systems (Table 3). The Himalstein classification (1973) stratifies MKS into three types according to tracheobronchial dilation severity [31], whereas Payandeh’s framework (2015) derives from a meta-analysis of 365 MKS cases across 166 studies, emphasizing its etiopathogenetic heterogeneity [10]. While the Himalstein system emphasizes structural abnormalities, the Payandeh framework incorporates etiopathogenetic considerations. This dichotomy underscores the necessity for clinicians to integrate multimodal clinical–radiological data for precise phenotyping.

**Table 3:** Comparative classification systems for MKS

Types	Descriptions
Anatomical Classification (Himalstein et al. [31])	
Type I	Relatively symmetrical, diffuse enlargement of the trachea and main bronchi
Type II	The enlargement is more obvious and has a bizarre or eccentric configuration. There may be diverticula
Type III	Diverticula or sacculations extend to the distal bronchi
Aetiological Classification (Payandeh et al. [10])	
Type 1A	Infants who have undergone FETO as a therapy for antenatally diagnosed severe congenital diaphragmatic hernia and developed TBM
Type 1B	Children or infants who developed TBM after prolonged intubation
Type 2A	Patients who developed TBM after multiple pulmonary infections
Type 2B	Patients who developed TBM after being diagnosed with pulmonary fibrosis
Type 3	Patients with TBM and evidence of extrapulmonary elastolysis
Type 4	Patients with TBM and no clear predisposing factors

*MKS* Mounier–Kuhn syndrome, *TBM* tracheobronchomegaly, *FETO* foetal endoscopic tracheal occlusion

Our study reveals that conservative management remains the cornerstone of MKS therapy, employed in 70.4% of cases (119/169). This aligns with current guidelines emphasizing symptom control through airway clearance and infection prevention in this anatomically driven disorder [11]. However, the modest rate of clinical stabilization (62.7%) and equivalent proportions of symptomatic improvement and treatment failure (8.3% each) underscore the limited therapeutic efficacy of conventional approaches. For asymptomatic MKS patients, therapeutic algorithms focus on infection prophylaxis, risk factor modulation, smoking cessation, and occupational irritant avoidance. During acute exacerbations with hypersecretion, strategies prioritize augmenting mucociliary clearance mechanisms via mucolytic agents and chest physiotherapy modalities (e.g., postural drainage). Targeted antibiotic regimens address superimposed infections guided by microbiological data [32].

Despite optimized management, progressive respiratory insufficiency may ensue, necessitating invasive interventions such as airway stenting or tracheobronchoplasty for severe dynamic collapse—although limited experience in MKS cohorts shows variable efficacy and frequent complications (infection, stent migration) [1, 33, 34]. Lung transplantation is reserved for patients whose end-stage respiratory failure is refractory to conventional therapies [35–37]. Notably, endoscopic laser ablation achieved sustained symptom remission in a 68-year-old MKS patient, demonstrating novel therapeutic potential [38]. The low utilization of ventilator-assisted support (11.2%) and surgery (10.1%) may reflect either under-recognition of advanced disease stages or reluctance to escalate care in a condition with no curative options. Notably, the 1:1 ratio between improvement and failure signals critical heterogeneity in treatment response, potentially tied to variations in tracheobronchial dilation severity or comorbid burden.

Perioperative airway management in MKS requires meticulous multidisciplinary planning. Surgical anaesthesia or prolonged positive-pressure ventilation mandates comprehensive otolaryngological–anaesthesiological evaluation [39]. Subglottic cuff placement with leak-controlled inflation optimizes tidal volume maintenance during intubation, which is complemented by laryngeal mask airways or oropharyngeal packing to prevent air leakage [40–42]. Modified laryngotracheal separation techniques enable high-pressure ventilation in neonates with tracheobronchomegaly and severe bronchopulmonary dysplasia [43]. Postprocedural monitoring of cuff pressures remains critical to balance tracheal seal efficacy and wall integrity preservation, as illustrated by Patient 1’s air leakage complications [44]. Therapeutic decisions require algorithm-driven selection on the basis of disease phenotype, pulmonary reserve, mechanical ventilation needs, and airway stability parameters.

## Conclusion

An integrated analysis of 169 literature-reported cases and 5 institutional cases established four epidemiologic features of MKS: (1) male predominance (male-to-female ratio 5.5:1); (2) tracheobronchial dilation (average tracheal diameter 34.3 mm); (3) frequent comorbidities, including bronchiectasis (71.6%) and tracheal diverticulosis (67.5%); and (4) chief complaints of cough (64.5%) and recurrent respiratory infections (57.4%). Despite advances in diagnostic imaging modalities dominating the recent research focus, significant diagnostic delays persist in clinical practice, as demonstrated by a median diagnostic interval of 3.0 years (IQR: 0.25–20.0 years). While the United States, European nations, and China drive research productivity, therapeutic strategies remain predominantly conservative (70.4%) with limited evidence-based innovation. These findings highlight the urgent need for standardized diagnostic protocols and international consortia to address the translational gap between imaging-driven detection and personalized therapeutic development in this underrecognized syndrome.

## Supplementary Information

Supplementary Material 1

Supplementary Material 2

Supplementary Material 3

## Data Availability

The datasets used during and/or analysed during the current study are available from the corresponding author upon reasonable request.

## Abbreviations

MKS: Mounier–Kuhn syndrome
TBM: Tracheobronchomegaly
CT: Computed tomography
