# Supplementary figures and images for: Mounier–Kuhn syndrome: a tripartite analysis bridging clinical epidemiology, imaging evolution, and global research landscapes
Source: Orphanet J Rare Dis. 2025 May 19;20:238. doi: 10.1186/s13023-025-03745-w (PMC12087173; doi:10.1186/s13023-025-03745-w)

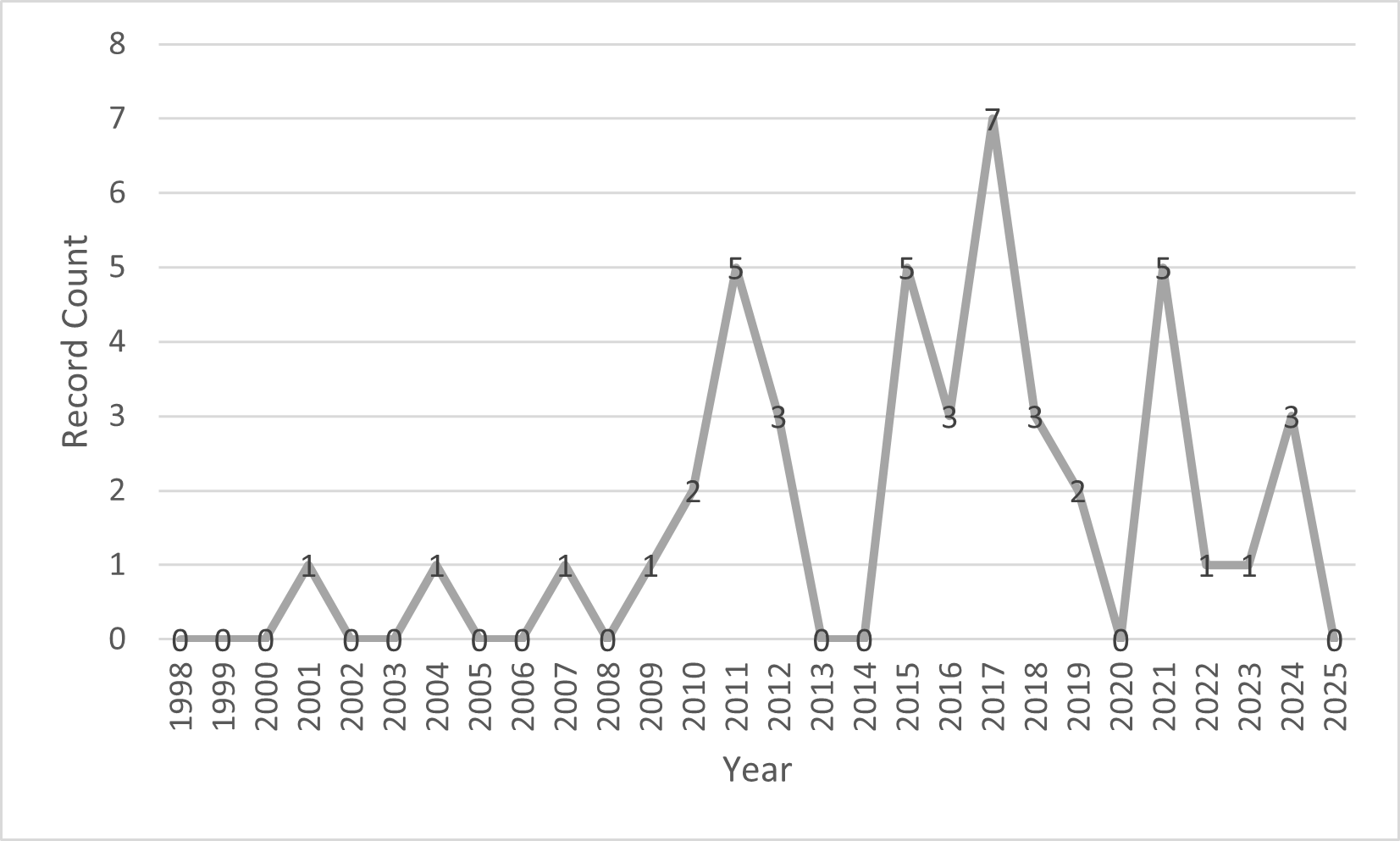

Supplement: Supplementary file 1 — Supplementary Material 1 [file 13023_2025_3745_MOESM1_ESM.png]

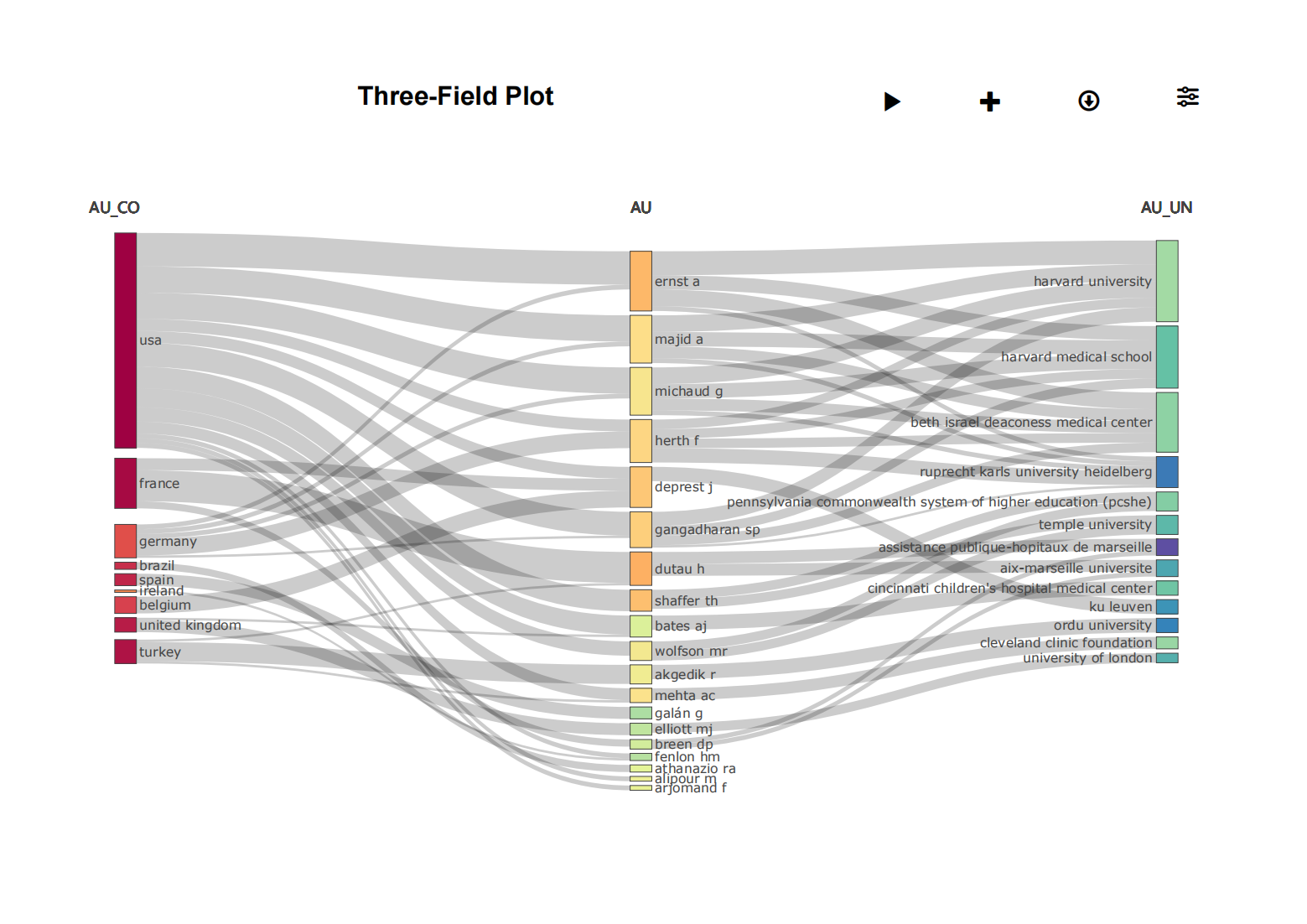

Supplement: Supplementary file 2 — Supplementary Material 2 [file 13023_2025_3745_MOESM2_ESM.tif]
