# Supplementary material for: Mounier–Kuhn syndrome: a tripartite analysis bridging clinical epidemiology, imaging evolution, and global research landscapes
Source: Orphanet J Rare Dis. 2025 May 19;20:238. doi: 10.1186/s13023-025-03745-w (PMC12087173; doi:10.1186/s13023-025-03745-w)
Supplement: Supplementary file 3 — Supplementary Material 3 [file 13023_2025_3745_MOESM3_ESM.doc]

| Rank | Institutions | Country | Frequency | Percentage(%) |
| --- | --- | --- | --- | --- |
| 1 | HARVARD UNIVERSITY | USA | 9 | 3.29 |
| 2 | BETH ISRAEL DEACONESS MEDICAL CENTER | USA | 6 | 2.19 |
| 3 | PENNSYLVANIA COMMONWEALTH SYSTEM OF HIGHER EDUCATION PCSHE | USA | 6 | 2.19 |
| 4 | US DEPARTMENT OF VETERANS AFFAIRS | USA | 6 | 2.19 |
| 5 | VETERANS HEALTH ADMINISTRATION VHA | USA | 6 | 2.19 |
| 6 | AIX MARSEILLE UNIVERSITE | France | 5 | 1.83 |
| 7 | CLEVELAND CLINIC FOUNDATION | USA | 5 | 1.83 |
| 8 | TEMPLE UNIVERSITY | USA | 5 | 1.83 |

**Supplementary Table 1** Number of publications by global research institution

| **Publication Titles** | **Frequency** | IF | JCR |
| --- | --- | --- | --- |
| CHEST | 35 | 9.5 | Q1 |
| AMERICAN JOURNAL OF RESPIRATORY AND CRITICAL CARE MEDICINE | 30 | 19.3 | Q1 |
| AMERICAN JOURNAL OF ROENTGENOLOGY | 8 | 4.7 | Q1 |
| ANESTHESIOLOGY | 5 | 9.1 | Q1 |
| ARCHIVOS DE BRONCONEUMOLOGIA | 5 | 8.7 | Q1 |
| REVUE DES MALADIES RESPIRATOIRES | 5 | 0.5 | Q4 |
| THORAX | 5 | 9 | Q1 |
| PEDIATRIC RADIOLOGY | 4 | 2.1 | Q2 |
| RESPIRATION | 4 | 3.5 | Q2 |
| REVISTA CLINICA ESPANOLA | 4 | 2.3 | Q2 |

**Supplementary Table 2** The top 10 journals contributing to the highest volume of publications on MKS research from 1962 to 2025. Abbreviations：IF，Impact Factor；JCR，Journal Citation Reports.

| Rank | Author | Highly cited documents | Source | JCR | IF | Year | Otal cite |
| --- | --- | --- | --- | --- | --- | --- | --- |
| 1 | Carden KA, Boiselle PM, Waltz DA, et al | Tracheomalacia and tracheobronchomalacia in children and adults: an in-depth review | Chest | Q1 | 9.5 | 2005 | 463 |
| 2 | Thiberville L, Moreno-Swirc S, Vercauteren T, et al | In vivo imaging of the bronchial wall microstructure using fibered confocal fluorescence microscopy | Am J Respir Crit Care Med | Q1 | 19.3 | 2007 | 242 |
| 3 | [Katz I, Levine M, Herman P](https://webofscience.clarivate.cn/wos/alldb/general-summary?queryJson=%5B{"rowBoolean":null,"rowField":"AU","rowText":"KATZ, IRA"}%5D&eventMode=oneClickSearch) | Tracheobronchiomegaly. The Mounier-Kuhn syndrome | Am J Roentgenol Radium Ther Nucl Med | Q1 | 4.7 | 1962 | 130 |

**Supplementary Table 3** List of highly cited papers on MKS in the Web of Science database published from 1962 to 2025. Abbreviations：IF，Impact Factor；JCR，Journal Citation Reports.
